# An Interpretable Belief Rule-Based Fault Diagnosis Method for Complex Equipment Considering Linguistic Fuzzy Information

**DOI:** 10.3390/e28060674

**Published:** 2026-06-11

**Authors:** Kun Wang, Tao Wang, Zhijie Zhou, Zhichao Ming, Zheng Lian, Kejun Wang

**Affiliations:** 1 College of Combat Support, PLA Rocket Force University of Engineering, Xi’an 710025, China; 2 College of Computer Science and Information Engineering, Harbin Normal University, Harbin 150025, China; 3 College of Missile Engineering, PLA Rocket Force University of Engineering, Xi’an 710025, China

**Keywords:** fault diagnosis, belief rule base, linguistic fuzzy information, interpretability

## Abstract

To address the challenges of linguistic fuzziness, cognitive variability across fault modes, and the risk of model distortion during optimization, this paper proposes an interpretable belief rule-based fault diagnosis method for complex equipment considering linguistic fuzzy information. First, to address the difficulty experts face in providing precise probability values, an interval grey number table is constructed. By converting linguistic fuzzy information into interval grey representations, the approach quantifies the uncertainty inherent in expert judgments while fully preserving the boundary information of the underlying knowledge. Second, recognizing that expert familiarity varies across different fault modes, a certainty degree fusion method is introduced. This method utilizes fusion weights to mitigate the interference of low-confidence evidence during rule generation. Finally, an interpretable parameter optimization method featuring dynamic knowledge anchoring is designed to constrain model parameters within the reasonable bounds defined by expert knowledge. Validation on an electromechanical actuator demonstrates that the proposed method not only achieves superior diagnostic performance but also ensures model usability and interpretability in practical engineering applications.

## 1. Introduction

With the rapid development of modern industrial technology, complex electromechanical equipment is evolving towards larger size, higher intelligence and better integration [1,2]. Such equipment features intricate structures and strong coupling among components. When a critical component fails, the consequences can include production downtime, substantial economic losses, and even major safety incidents [3]. Consequently, conducting timely and effective fault diagnosis after a failure is essential for making precise maintenance decisions in engineering practice.

In complex engineering scenarios, the failure modes of equipment are often difficult to fully characterize, and valuable fault data remain extremely scarce. Under such conditions, relying solely on model-based or purely data driven approaches is rarely effective [4,5,6]. Methods that integrate expert knowledge with limited data have thus emerged as a promising alternative [7,8]. However, expert knowledge is inherently subjective and imprecise. Due to cognitive limitations and incomplete field information, experts often struggle to assign precise probabilities when describing causal relationships between fault features and fault modes. Instead, they tend to rely on qualitative linguistic expressions, such as “relatively high temperature” or “probability between 50 and 90” [9,10,11]. Existing research on quantifying such uncertain information has largely focused on two main paradigms: fuzzy set theory [12,13] and probabilistic statistical inference [14,15]. Fuzzy logic methods typically convert qualitative linguistic variables into quantitative numerical forms using empirically defined membership functions, such as triangular [16], trapezoidal, or Gaussian functions [17]. However, these approaches require precise specification of the membership functions. When sufficient data are lacking, imposing a particular functional form can make the model parameters highly dependent on the subjective choices and predefined criteria of the designer [18]. In contrast, probabilistic reasoning methods such as Bayesian networks and Dempster–Shafer evidence theory are theoretically rigorous, but their modeling process generally demands complete prior information. Effective inference often requires constructing conditional probability tables or prior probability distributions with precisely determined parameters [19,20], which is particularly challenging in fault diagnosis scenarios characterized by limited information. Therefore, achieving reliable diagnosis from fault features to fault modes calls for a reasoning framework that can effectively accommodate imprecise information while maintaining a high degree of interpretability.

As artificial intelligence technologies are increasingly deployed in high reliability domains such as military systems, aviation, and healthcare, the transparency, interpretability, and trustworthiness of model decisions have become central factors limiting their practical adoption [21,22]. Compared with black box models like deep learning, the belief rule base (BRB) stands out for its strong interpretability and rigorous reasoning framework. First introduced by Yang et al. in 2006 [23], BRB uses an intuitive “IF-THEN” rule structure that naturally incorporates qualitative expert knowledge, quantifies uncertainty in the form of belief distributions, and achieves logically consistent nonlinear mapping through the evidential reasoning algorithm [24]. Its inference process is highly interpretable, which has led to its application in areas such as system health management, safety risk assessment, and medical diagnosis [25,26,27]. In particular, for fault diagnosis in complex high precision equipment, BRB offers distinct advantages in handling multi-source heterogeneous uncertainty [28]. Although BRB has a relatively mature theoretical framework and uncertainty reasoning capability, it still faces some challenges when modeling and optimizing for the above-mentioned complex uncertainty scenarios. First, traditional rule-based reasoning frameworks usually rely on a rule base manually constructed by experts in advance according to a set rule structure. The reasoning process is relatively clear and interpretable, but the performance of the diagnostic model highly depends on the completeness and reliability of the initial rules [29]. For complex equipment fault diagnosis, experts are often more likely to provide directional judgments of single features on fault types, but it is difficult to directly give complete belief rules and precise belief degrees [30,31]. Therefore, how to transform fragmented and uncertain expert knowledge into reliable initial rules is still a key issue in rule-based reasoning models. Second, due to differences in the occurrence frequency, sample accumulation, and expert experience of different fault types, experts’ cognitive certainty about different fault modes is not completely consistent. If this difference is ignored in the evidence fusion process, knowledge with low certainty degree or strong ambiguity may be over-utilized, thereby affecting the reliability of diagnostic results [32]. In addition, when using limited historical data for parameter optimization, existing BRB optimization methods usually adjust parameters such as rule weights, attribute weights, and belief degrees based on a given initial rule structure to improve diagnostic accuracy [33]. However, when the optimization objective is mainly driven by data fitting accuracy, the optimized parameters may deviate from the empirical range or physical constraints given by experts, thereby weakening the original interpretability advantage of rule-based reasoning methods [34,35].

To address these challenges, this paper proposes a fault diagnosis method based on an interpretable belief rule-based that accounts for linguistic fuzzy information. The method aims to establish an interpretable pathway from expert knowledge to a high performance diagnostic model.

(1) **A linguistic fuzzy information representation method based on interval grey numbers:** By constructing an interval grey number table, the linguistic judgments of experts regarding the relationships between fault features and fault modes are transformed into interval grey numbers. This preserves the uncertain boundary information in expert judgments and provides a quantitative foundation for subsequent evidence extraction and rule generation.
(2) **A certainty degree-weighted evidence fusion and initial rule generation method:** Considering the difference of expert cognitive certainty under different fault modes, the role of evidence in the fusion process is adjusted by certainty weighting to reduce the interference of low certainty evidence on the generation of initial rules.
(3) **An interpretable parameter optimization method with dynamic knowledge anchoring:** It uses expert knowledge to constrain and guide parameter search in the data-driven optimization process, prevents optimization results from deviating too much from expert experience and physical meaning, and improves fault diagnosis performance while maintaining the interpretability of BRB. Finally, a fault diagnosis experiment of an electromechanical actuator is carried out to verify the effectiveness and robustness of the proposed method.

In terms of methodology, the proposed approach can be regarded as an extension of uncertainty handling and interpretability preservation in the framework of BRB. As shown in Table 1, the proposed method differs from existing rule-based reasoning frameworks mainly in terms of knowledge representation, initial rule construction approaches, knowledge uncertainty handling, and parameter optimization schemes. Traditional BRB fault diagnosis methods usually assume that experts can provide relatively clear belief degree or relatively complete initial rules. This method does not directly require experts to construct a complete rule base, but starts from linguistic fuzzy expert judgment, converts it into interval grey number representation, and further generates initial belief rules through certainty weighted evidence fusion. At the same time, to solve the problem that data-driven parameter optimization may cause rule parameters to deviate from expert knowledge and physical meaning, this paper introduces a dynamic knowledge anchoring mechanism to constrain and guide the optimization process. Therefore, the main difference of the proposed approach does not lie in changing the basic reasoning mechanism of BRB but in constructing a complete modeling path from linguistic fuzzy information representation and certainty degree-based rule generation to interpretable parameter optimization.

The remainder of this paper is organized as follows. Section 2 introduces the fundamental theory of the belief rule base and describes the problem addressed in this paper. Section 3 elaborates on the core fault diagnosis method proposed in this paper. Section 4 presents the interpretable parameter optimization method with dynamic knowledge anchoring. Section 5 validates the effectiveness of the proposed approach in terms of diagnostic accuracy and interpretability preservation through experiments conducted on an electromechanical actuator (EMA). Section 6 concludes the paper.

## 2. Basics and Problem Formulation

### 2.1. Basics of Belief Rule Base

A belief rule base (BRB) is an expert system modeling approach that uses evidential reasoning as its inference engine. Its core principle lies in transforming prior knowledge of system faults and expert experience into structured belief rules. By representing the uncertainty of diagnostic results through belief degrees, BRB effectively addresses common challenges in fault diagnosis such as incomplete information and multi-attribute decision making.

Belief rules are used to establish the mapping between fault features and system working states. In fault diagnosis, a diagnostic framework is first defined. Let $F=\{F_{1},F_{2},\cdots ,F_{N}\}$ be the set consisting of *N* working states, and let $X=\{x_{1},x_{2},\cdots ,x_{M}\}$ be the set of fault features. The *k*th rule can then be expressed as follows:

(1) $$\begin{array}{cc} {Rl}_{k}:\; & \mathrm{If}\;x_{1}\;\mathrm{is}\;A_{1}^{k}\wedge x_{2}\;\mathrm{is}\;A_{2}^{k}\wedge \cdots \wedge x_{M}\;\mathrm{is}\;A_{M}^{k},\mathrm{then}\;\left\{\left(F_{1},\xi_{k,1}\right),\left(F_{2},\xi_{k,2}\right),\cdots ,\left(F_{N},\xi_{k,N}\right)\right\}\\ \; & \mathrm{with}\;\mathrm{rule}\;\mathrm{weight}\;\omega_{k}\;\mathrm{and}\;\mathrm{fault}\;\mathrm{feature}\;\mathrm{weights}\;\varepsilon_{1},\varepsilon_{2},\cdots ,\varepsilon_{M} \end{array}$$

The antecedent of each rule is composed of reference states or fuzzy sets corresponding to the input fault features. Let $A_{i}^{k}\in \left\{A_{i,j},i=1,2,\cdots M;j=1,2,\cdots ,J_{i}\right\}$ denote the reference value of the *i*th fault feature in the *k*th rule. The consequent is expressed as a belief distribution over the working state, where $\xi_{k,j}$ represents the belief degree assigned to working state $F_{j}$ in the *k*th rule, satisfying $\sum_{j=1}^{N}\xi_{k,j}\leq 1$. Each rule is assigned a rule weight $\omega_{k}\in \left[0,1\right]$ that reflects its importance, and each fault feature $\varepsilon_{i}\in \left[0,1\right]$ is assigned a fault feature weight that indicates its influence on the activation degree of the rules.

### 2.2. Description of the Problems

**Problem 1: Uncertainty in linguistic fuzzy information.** When leveraging expert knowledge for fault diagnosis, experts typically cannot provide precise probability values for the relationships between fault features and working states. Instead, they rely on experience to offer a range of possibilities or qualitative statements, which inherently involve uncertainty. Such expressions often take the form of vague descriptions such as “the probability lies within a certain range”, making it difficult to pinpoint exact numerical values. Therefore, transforming this type of linguistic fuzzy information into a normalized representation suitable for subsequent mathematical computation and fusion constitutes the primary challenge in effectively utilizing expert knowledge. This issue can be described as follows:

(2) $$NorR=Z\left(L;F,X,A\right)$$

where $F=\{F_{1},F_{2},\cdots ,F_{N}\}$ denotes the set of working states, $X=\{x_{1},x_{2},\cdots ,x_{M}\}$ denotes the set of fault features, and $A=\{A_{i,j},i=1,2,\cdots M;j=1,2,\cdots ,J_{i}\}$ denotes the set of reference levels. Z(·) is a mapping function that transforms the linguistic fuzzy knowledge *L* into a normalized representation NorR.

**Problem 2: Cognitive variability across fault modes.** Because different faults occur at varying frequencies, expert may have differing levels of familiarity with each fault mode. Traditional evidential reasoning rules compress the multidimensional certainty associated with a single piece of evidence across multiple fault modes into a single reliability value. This fusion approach treats highly confident diagnostic indications and low confidence speculations equally. Within an information fusion framework, addressing this cognitive variability, which varies by fault mode, represents a key challenge in improving the reliability and accuracy of diagnostic results. This issue can be described as follows:

(3) $$\phi =\psi \left(E_{a},Q\right)$$

where $E_{a}$ denotes expert knowledge that exhibits cognitive variability, *Q* denotes fault data, ψ(·) is the fusion function, and ϕ represents the fused result.

**Problem 3: Interpretable model optimization.** When optimizing model parameters based on data, it is easy to cause the optimization results to seriously deviate from the initial knowledge provided by experts. Such deviation not only diminishes the prior value of expert knowledge but also undermines the physical meaning of the rule base. Consequently, the model may achieve a good fit to the data yet lose interpretability by violating engineering common sense, thereby reducing its credibility in practical applications. This issue can be described as follows:

(4) $$\max_{\Theta }P\left(\Theta \right)\;s.t.\;Y\left(\Theta \right)$$

where Θ denotes the set of parameters, P(·) represents the model performance metric, and Y(·) is a constraint function that measures the consistency between the parameters and the expert knowledge.

## 3. Fault Diagnosis Model Based on a Belief Rule Base with Linguistic Fuzzy Information

**Figure 1:**
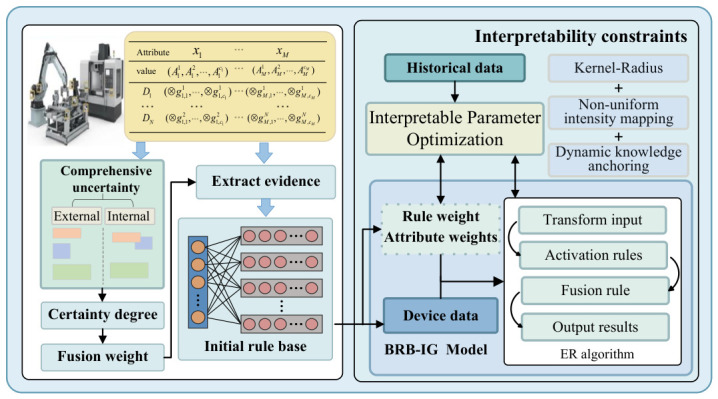
The process of the method proposed in this paper.

This section presents a fault diagnosis model based on a belief rule base with interval grey numbers (referred to as BRB-IG), which addresses Problems 1 and 2. Consistent with the methodological positioning described above, this section focuses on the interval grey representation of linguistic fuzzy information and the certainty-weighted evidence extraction and fusion process. Specifically, Section 3.1 constructs the interval grey number tables to achieve the formalized representation of uncertain expert knowledge; Section 3.2 completes the certainty-weighted evidence extraction and fusion, and generates the initial belief rules. Section 3.3 elaborates on the reasoning process of BRB. Figure 1 illustrates the main flowchart of the method proposed in this paper.

### 3.1. Construction of the Interval Grey Number Table

In fault diagnosis for complex equipment, expert knowledge is often linguistically fuzzy and difficult to express as a single numerical value. Forcing such knowledge into a precise value may lead to information loss and bias. The interval grey number, a concept from grey system theory, is capable of representing information that is “partially known and partially unknown” [36,37]. Accordingly, this paper proposes an interval grey number table to characterize such knowledge and to serve as the basis for constructing the initial rule base.

When constructing the interval gray number table, the expert assigns an interval gray number $⊗g_{m,i}^{n}$ to each working state $F_{n}$ and each reference level $A_{m}^{i}$ for a given fault feature $x_{m}$ according to the empirical knowledge and the physical mechanism. The general expression is as follows:

(5) $$P\left(x_{m}=A_{m}^{i}|F_{n}\;happened\right)=⊗g_{m,i}^{n}$$

where $⊗g_{m,i}^{n}\in \left[l_{m,i}^{n},u_{m,i}^{n}\right]$, which satisfies $l_{m,i}^{n},u_{m,i}^{n}\in \left[0,1\right]$ and $l_{m,i}^{n}\leq u_{m,i}^{n}$. This interval grey number represents the probability of the fault feature falling within the reference level when the operating state $F_{n}$ occurs. Both the upper and lower bounds lie within the [0,1] probability scale. To provide semantic reference, 0 is defined as almost impossible, 0.4 as possible, 0.7 as relatively likely, and 1 as almost certain. The measure of the interval grey number can be used to roughly measure the uncertainty of the expert’s judgment; a larger measure indicates that the expert is more uncertain, while a smaller measure indicates that the expert’s judgment is relatively certain. When converting linguistic fuzzy information into interval grey numbers, experts first make an initial estimation of the probability of the feature occurring under a specific reference level, based on their own experience, equipment mechanisms, and the historical data they master; subsequently, the interval length is determined according to the degree of certainty of the judgment, thereby obtaining the final interval grey number. This method makes the quantification of expert knowledge possess operability and traceability, providing a reliable foundation for constructing the initial rule base. The structure of the interval gray number table provided by the experts is shown in Table 2.

For a specific working state $F_{n}$ and a given fault feature $x_{m}$, the set of interval grey numbers corresponding to all reference levels $\left\{A_{m}^{1},A_{m}^{2},\cdots ,A_{m}^{J_{m}}\right\}$ forms an interval grey array, denoted as

(6) $$G_{m}^{n}=\left\{⊗g_{m,1}^{n},⊗g_{m,1}^{n},\cdots ,⊗g_{m,J_{m}}^{n}\right\}$$

where $J_{m}$ denotes the number of reference values for the *m*th fault feature. This array serves as the fundamental unit for subsequent knowledge processing and computation.

**Remark 1.** 
*Traditional fuzzy rule methods usually rely on preset membership functions, while standard belief rule base methods often require experts to provide relatively explicit belief degrees or initial rules. In contrast, interval grey numbers can preserve the upper and lower bound information of expert judgments, allowing linguistic fuzzy knowledge to enter the rule generation process in an interval form.*

### 3.2. Certainty Degree-Based Evidence Extraction and Fusion

To address the issue of differences in expert cognitive levels under different fault modes, this paper constructs a certainty degree-based evidence extraction and fusion method. Its core idea is to establish a complete transformation chain from interval grey number knowledge to initial rules. First, the uncertainty inherent in expert judgments is measured from the perspectives of both measure and inter-interval distinguishability. Then, the comprehensive uncertainty is converted into a certainty degree oriented toward specific working states and reference levels, which serves to characterize the reliability of the judgment. Finally, in the evidence fusion stage, the certainty degree is introduced into the fusion weights, allowing the evidence to participate in rule generation according to its certainty. Through this mechanism, the high-certainty portion of the evidence makes a larger contribution to the generation of consequent certainty, while the low-certainty portion is not directly discarded; instead, its influence is reduced through weight decay. This preserves useful expert knowledge while minimizing the interference of vague or low-reliability judgments on the construction of the rule base.

(1) Comprehensive Uncertainty Measurement

For an interval grey number, its uncertainty originates not only from its own measure but also from its relative relationship with other interval grey numbers in the same group. The measure of an interval grey number reflects the internal uncertainty of the expert’s judgment. Additionally, the degree of overlap or distance relationship with other interval grey numbers in the same group reflects the external uncertainty of that reference level relative to others. When two intervals overlap significantly or are close in distance, it indicates that the distinguishability between their corresponding reference levels is low, making the indicative role of the judgment more prone to ambiguity. Therefore, this paper combines internal and external uncertainty to define the comprehensive uncertainty. For a given interval grey number $⊗g_{m,i}^{n}=\left[l_{m,i}^{n},u_{m,i}^{n}\right]$, the comprehensive uncertainty is defined as follows:

(7) $$U_{m,i}^{n}=w_{m,i}^{n}\times \left[\frac{1}{\left|G_{m}^{n}\right|-1}\sum_{j\neq i}^{\left|G_{m}^{n}\right|}R\left(⊗g_{m,i}^{n},⊗g_{m,j}^{n}\right)\right]$$

where $w_{m,i}^{n}=u_{m,i}^{n}-l_{m,i}^{n}$ is a measure of the interval grey number, representing internal uncertainty. $\left|G_{m}^{n}\right|$ denotes the total number of interval grey numbers in the group $G_{m}^{n}$ to which the given interval grey number belongs. The relationship measurement function $R\left(⊗g_{m,i}^{n},⊗g_{m,j}^{n}\right)$ is used to evaluate the relative relationship between $⊗g_{m,i}^{n}$ and another interval grey number $⊗g_{m,j}^{n}$ within the same group, thereby reflecting external uncertainty. $R\left(⊗g_{m,i}^{n},⊗g_{m,j}^{n}\right)$ is defined in two cases depending on whether the two intervals overlap:

(8) $$R\left(⊗g_{m,i}^{n},⊗g_{m,j}^{n}\right)=\left\{\begin{array}{c} H\left(⊗g_{m,i}^{n},⊗g_{m,j}^{n}\right),\min\left(u_{m,i}^{n},u_{m,j}^{n}\right)>\max\left(l_{m,i}^{n},l_{m,j}^{n}\right)\\ 1-\frac{D(⊗g_{m,i}^{n},⊗g_{m,j}^{n})}{S},\min\left(u_{m,i}^{n},u_{m,j}^{n}\right)\leq \max\left(l_{m,i}^{n},l_{m,j}^{n}\right) \end{array}\right.$$

(a) When two intervals overlap, the Jaccard coefficient $H\left(⊗I_{i},⊗I_{j}\right)$ is used to measure the degree of overlap. It is defined as the ratio of the length of the overlap to the length of the union of the two intervals:

(9) $$H\left(⊗g_{m,i}^{n},⊗g_{m,j}^{n}\right)=\frac{\left|⊗g_{m,i}^{n}\cap ⊗g_{m,j}^{n}\right|}{\left|⊗g_{m,i}^{n}\cup ⊗g_{m,j}^{n}\right|}=\frac{O_{length}\left(⊗g_{m,i}^{n},⊗g_{m,j}^{n}\right)}{w_{m,i}^{n}+w_{m,j}^{n}-O_{length}\left(⊗g_{m,i}^{n},⊗g_{m,j}^{n}\right)}$$

(10) $$O_{length}\left(⊗g_{m,i}^{n},⊗g_{m,j}^{n}\right)=\max\left(0,\min\left(u_{m,i}^{n},u_{m,j}^{n}\right)-\max\left(l_{m,i}^{n},l_{m,j}^{n}\right)\right)$$

where $O_{length}\left(⊗g_{m,i}^{n},⊗g_{m,j}^{n}\right)$ denotes the length of the overlap.

(b) When the two interval grey numbers are non-overlapping, $D\left(⊗g_{m,i}^{n},⊗g_{m,j}^{n}\right)$ represents the distance between the two intervals, and *S* is a normalization factor defined as the total span of the interval group $G_{m}^{n}$, calculated as follows:

(11) $$D\left(⊗g_{m,i}^{n},⊗g_{m,j}^{n}\right)=\max\left(l_{m,i}^{n},l_{m,j}^{n}\right)-\min\left(u_{m,i}^{n},u_{m,j}^{n}\right)$$

(12) $$S=\max\left(\left\{u_{m,k}^{n}\left|⊗g_{m,k}^{n}\in G_{m}^{n}\right.\right\}\right)-\min\left(\left\{l_{m,k}^{n}\left|⊗g_{m,k}^{n}\in G_{m}^{n}\right.\right\}\right)$$

(2) Conversion to Confidence Degree

After obtaining the comprehensive uncertainty $U_{m,i}^{n}$, it needs to be further converted into a certainty degree, providing a positive indicator that characterizes the reliability of the evidence for the subsequent evidence fusion process. Since comprehensive uncertainty itself reflects the degree of fuzziness in the judgment, a negative correlation should exist between the two. The transformation formula is as follows:

(13) $$C_{m,i}^{n}={\left(1-U_{m,i}^{n}\right)}^{\tau }$$

The certainty degree $C_{m,i}^{n}$ is used to measure the expert’s level of certainty regarding the judgment that “given the current state of the fault feature $x_{m}$, it points to a particular fault $F_{n}$”. τ>0 is the sensitivity coefficient, which serves to adjust the response intensity of the certainty degree to changes in uncertainty. When τ>1, the certainty degree decreases more rapidly with the increase in comprehensive uncertainty, which is suitable for diagnostic scenarios that are relatively sensitive to low-certainty evidence and require strict suppression of fuzzy judgments; when 0<τ<1, the transformation process is relatively gentle, allowing evidence with a certain level of uncertainty to retain its contribution. A larger certainty degree indicates a higher confidence of the expert in this interval judgment. This transformation not only achieves the mapping from uncertain information to reliability measures but also provides a basis for the adaptive suppression of low-certainty evidence in the subsequent fusion process.

(3) Evidence Extraction

To convert the interval grey numbers into the input format required by the evidential reasoning algorithm, a whitening process is applied to the interval grey numbers. A confidence coefficient $\mu \in \left[0,1\right]$ is introduced to represent the expert’s level of confidence in their own judgment, where 0 indicates complete confidence, and 1 indicates no confidence. Based on this, the interval grey numbers are adjusted to obtain the basic probability assignments

(14) $${\tilde{p}}_{m,i}^{n}=\frac{l_{m,i}^{n}+u_{m,i}^{n}}{2}\times \left(1-w_{m,i}^{n}\mu \right)+\frac{1}{\left|G_{m}^{n}\right|}\times w_{m,i}^{n}\mu$$

To ensure that all basic probabilities within the same interval grey array form a complete probability distribution, the ${\tilde{p}}_{m,i}^{n}$ must be normalized

(15) p^m,in=p˜m,inp˜m,in∑j=1Gmnp˜m,jn∑j=1Gmnp˜m,jn

At this point, for any given set of conditions in the interval grey number table, namely the working state $F_{n}$, fault feature $x_{m}$, and reference level $A_{m}^{i}$, a corresponding piece of evidence $E_{m,i}$ can be extracted and expressed as follows:

(16) $$\begin{array}{cc} E_{m,i}:\; & \mathrm{If}\;x_{m}\;\mathrm{is}\;A_{m}^{i},\mathrm{then}\;\left\{\left(F_{1},p_{m,i}^{1}\right),\left(F_{2},p_{m,i}^{2}\right),\cdots ,\left(F_{N},p_{m,i}^{N}\right)\right\}\\ \; & \mathrm{with}\;\mathrm{certainty}\;\mathrm{degrees}\;\left\{C_{m,i}^{1},C_{m,i}^{2},\cdots ,C_{m,i}^{N}\right\}\;\mathrm{and}\;\mathrm{fault}\;\mathrm{feature}\;\mathrm{weight}\;\varepsilon_{m} \end{array}$$

where pm,in=p^m,inp^m,in∑n=1Np^m,in∑n=1Np^m,in. The entire set of evidence forms the foundation for subsequent model construction, providing quantifiable inputs for building the belief rule base with minimal loss of expert knowledge.

(4) Initial rule base construction through certainty-weighted evidence fusion

After completing the evidence extraction, it is necessary to fuse the evidence corresponding to multiple fault features into a complete belief rule. Traditional evidence reasoning methods usually employ a single reliability to characterize the overall credibility of a piece of evidence. However, in actual diagnostic processes, due to variations in the occurrence frequency of different fault modes and the degree of expert familiarity, the reliability of expert judgments under different fault modes is inconsistent. Furthermore, while the support degree of the same evidence for different fault modes can be represented by basic probability assignment, its reliability should also possess fault mode dependency. Therefore, this part introduces the certainty degree into the fusion weights. During the process of fusing multiple pieces of evidence corresponding to a specific antecedent combination into a single belief rule, fusion weights are assigned to each piece of evidence based on the expert’s certainty degree for different fault modes, thereby differentially allocating their weights in the final rule generation. The fusion weight is calculated as follows:

(17) $$\upsilon_{m,i}^{n}=\frac{\varepsilon_{m}}{1+\varepsilon_{m}-C_{m,i}^{n}}$$

where the fusion weight is jointly determined by the fault feature weight $\varepsilon_{m}$ and the certainty degree $C_{m,i}^{n}$. Among them, $\varepsilon_{m}$ reflects the importance of the fault feature $x_{m}$ in the diagnosis, while the certainty degree $C_{m,i}^{n}$ reflects the local reliability of the evidence under a specific fault mode and reference level. It can be seen from the equation that when the certainty degree $C_{m,i}^{n}$ increases, the fusion weight increases accordingly, indicating that the judgment of the evidence regarding the fault mode is relatively reliable and should play a stronger role in the generation of the consequent belief. Conversely, when $C_{m,i}^{n}$ is small, the denominator increases, and the fusion weight decreases, thereby weakening the influence of the evidence on the corresponding fault mode. Consequently, low-certainty evidence is not directly eliminated but is suppressed through weight decay, which avoids excessive interference of low-certainty speculative information on the final belief distribution while preserving the valid expert knowledge it may contain.

After obtaining the differentiated fusion weights $\upsilon_{m,i}^{n}$ for each fault mode, it yields the belief degree for fault mode $F_{j}$ in the *k*th rule. Taking the first rule as an example:

(18) $$\begin{array}{cc} R_{1}: & \mathrm{If}\;x_{1}\;\mathrm{is}\;A_{1}^{1}\wedge x_{2}\;\mathrm{is}\;A_{2}^{1}\wedge \cdots \wedge x_{m}\;\mathrm{is}\;A_{m}^{1},\mathrm{then}\;\left\{\left(F_{1},\xi_{1,1}\right),\left(F_{2},\xi_{2,1}\right),\cdots ,\left(F_{N},\xi_{N,1}\right)\right\}\\ \; & \mathrm{with}\;\mathrm{rule}\;\mathrm{weight}\;\omega_{1}\;\mathrm{and}\;\mathrm{fault}\;\mathrm{feature}\;\mathrm{weights}\;\left\{\varepsilon_{1},\varepsilon_{2},\cdots ,\varepsilon_{M}\right\} \end{array}$$

The belief degree for working state $F_{j}$ in the first rule is obtained as follows:

(19) $$\xi_{j,1}=\frac{\prod_{m=1}^{M}\left(\upsilon_{m,1}^{n}p_{m,1}^{j}+1-\upsilon_{m,1}^{n}\sum_{n=1}^{N}p_{m,1}^{n}\right)-\prod_{m=1}^{M}\left(1-\upsilon_{m,1}^{n}\sum_{n=1}^{N}p_{m,1}^{n}\right)}{\sum_{n=1}^{N}\prod_{m=1}^{M}\left(\upsilon_{m,1}^{n}p_{m,1}^{n}+1-\upsilon_{m,1}^{n}\sum_{n=1}^{N}p_{m,1}^{n}\right)-\left(N-1\right)\prod_{m=1}^{M}\left(1-\upsilon_{m,1}^{n}\sum_{n=1}^{N}p_{m,1}^{n}\right)-\prod_{m=1}^{M}\left(1-\varepsilon_{m}\right)}$$

Based on the above calculations, an initial rule base can be constructed, providing a structured knowledge foundation for subsequent fault diagnosis.

**Figure 2:**
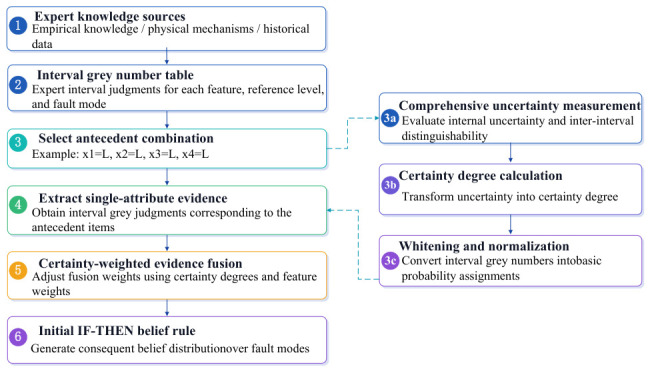
The generation process from expert knowledge to the initial IF-THEN belief rules.

The generation process from expert knowledge to the initial IF-THEN belief rules is shown in Figure 2. First, based on empirical knowledge, fault physical mechanisms, and historical data, experts make interval judgments on the relationship between different fault features, reference grades, and fault modes, forming an interval grey number table. Then, the comprehensive uncertainty is calculated from two aspects, intra-interval uncertainty and inter-interval discriminability, and further converted into a certainty degree, which is used to characterize evidence reliability. Subsequently, for a specific antecedent combination, the corresponding single-attribute evidence is extracted from the interval grey number table, and the interval grey numbers are converted into basic probability assignments through whitening and normalization processing. Finally, in the process of evidence fusion, the certainty degree and feature weights are jointly introduced into the fusion weights to generate the initial IF-THEN belief rule corresponding to the antecedent combination.

**Remark 2.** 
*Traditional BRB methods typically perform reasoning based on an existing rule base, whereas the proposed method first extracts evidence from experts’ linguistic fuzzy judgments and then generates initial belief rules through evidence fusion. Considering that experts’ degrees of confidence regarding different fault modes or judgment relationships may not be completely consistent, the certainty degree is introduced to characterize the trustworthiness of the corresponding evidence. By regulating the role of different pieces of evidence during the fusion process, this approach mitigates the impact of low-certainty evidence on the generation of initial rules.*

### 3.3. Inference of the Belief Rule Base

(1) Input Transformation

A transformation method based on rules and utilities is adopted to convert the observed values of each fault feature into a belief distribution. For the input $q_{i}$ of the *i*th attribute, the matching degrees with respect to the predefined reference values are calculated to obtain the corresponding belief degrees. This process uniformly represents numerical inputs in the form of a belief distribution. The transformation is expressed as follows:

(20) $$\alpha_{i,j}=\frac{A_{i}^{j+1}-q_{i}}{A_{i}^{j+1}-A_{i}^{j}},\;\;\;x_{i,j}\leq q_{i}\leq x_{i,j+1}$$

(21) $$\alpha_{i,j+1}=1-\alpha_{i,j},\;\;\;x_{i,j}\leq q_{i}\leq x_{i,j+1}$$

where $\alpha_{i,j}$ and $\alpha_{i,j+1}$ denote the belief degrees associated with the reference values $A_{i}^{j}$ and $A_{i}^{j+1}$, respectively.

(2) Rule Activation

Based on the belief distribution representation of the inputs, the activation weight of each rule is calculated. First, the overall matching degree of the *k*th rule is determined, which measures the degree to which the input information matches the antecedent of the rule.

(22) $$\alpha_{k}=\prod_{i=1}^{M}{\left(\alpha_{i}^{k}\right)}^{{\overset{-}{\varepsilon }}_{k,i}},\;\;\alpha_{i}^{k}\in \left\{\alpha_{i,j}\right\}$$

Next, the weights of the fault features are normalized.

(23) $${\overset{-}{\varepsilon }}_{k,i}=\frac{\varepsilon_{k,i}}{\max_{i=1,...,M}\left\{\varepsilon_{k,i}\right\}},so\;that\;0\leq {\overset{-}{\varepsilon }}_{k,i}\leq 1$$

Finally, the activation weight of the *k*th rule is calculated by incorporating the rule weight, reflecting the extent to which the rule is invoked during inference.

(24) $$\varpi_{k}=\frac{\omega_{k}\alpha_{k}}{\sum_{i=1}^{K}\omega_{i}\alpha_{i}}$$

(3) Rule Fusion

The analytical evidential reasoning algorithm is employed to combine the outputs of all activated rules, yielding the belief degrees $\vartheta_{j}$ corresponding to each fault mode. The final diagnostic result of the equipment is then output based on these belief degrees.

(25) $$\mu ={\left[\sum_{j=1}^{N}\prod_{k=1}^{K}\left(\varpi_{k}\xi_{j,k}+1-\varpi_{k}\sum_{i=1}^{T}\xi_{j,k}\right)-\left(N-1\right)\prod_{k=1}^{K}\left(1-\varpi_{k}\sum_{i=1}^{N}\xi_{j,k}\right)-\prod_{k=1}^{K}\left(1-\varpi_{k}\right)\right]}^{-1}$$

(26) $$\vartheta_{j}=\frac{\mu \times \left[\prod_{k=1}^{K}\left(\varpi_{k}\xi_{j,k}+1-\varpi_{k}\sum_{i=1}^{N}\xi_{j,k}\right)-\prod_{k=1}^{K}\left(1-\varpi_{k}^{w}\sum_{i=1}^{N}\xi_{j,k}\right)\right]}{1-\mu \times \left[\prod_{k=1}^{K}\left(1-\omega_{k}\right)\right]\;}$$

## 4. Interpretable Parameter Optimization with Dynamic Knowledge Anchoring

Aiming at the problems of excessive blindness, deviation from expert experience and even violation of physical knowledge in the parameter optimization of projection covariance matrix adaptive evolution strategy (P-CMA-ES), this paper proposes an interpretable parameter optimization method based on dynamic knowledge anchoring. This method restructures the search space and evolutionary trajectory, thereby ensuring the interpretability and robustness of the diagnostic model. The procedure consists of the following steps.

Step 1: Center–radius parameterization and parameter initialization.

To avoid the possibility of inverse differences (i.e., the lower bound exceeding the upper bound) when P-CMA-ES directly optimizes the upper and lower bounds of the interval grey numbers provided by experts, the proposed method transforms the representation into a center–radius space. The optimization vector is

(27) $$P={\left[a_{c},r,w,v\right]}^{T}$$

where $a_{c}=\left\{\left(l_{m,i}^{n}+u_{m,i}^{n}\right)/2,\;n=1,2,\cdots ,N;\;m=1,2,\cdots ,M;\;i=1,2,\cdots ,J_{m}\right\}$ denotes the center of the grey numbers, $r=\left\{w_{m,i}^{n}/2,\;n=1,2,\cdots ,N;\;m=1,2,\cdots ,M;\;i=1,2,\cdots ,J_{m}\right\}$ denotes the cognitive grey radius, and $w=\left\{\omega_{1},\omega_{2},\cdots \omega_{K}\right\}$ and $v=\left\{\varepsilon_{1},\varepsilon_{2},\cdots ,\varepsilon_{M}\right\}$ represent the fault feature weights and rule weights, respectively. The mean, step size, covariance matrix, and evolutionary paths are initialized, and hyperparameters such as population size and learning rate are set.

Step 2: Sampling and non-uniform intensity mapping.

To achieve a non-uniform search strategy that performs fine grained exploration near the values provided by experts and broad coverage at a distance, a nonuniform intensity mapping method is introduced. The standardized variation x∈[−1,1] generated for each individual is not directly applied to the physical parameters. Instead, it is transformed through a power mapping:

(28) $$\Delta P=\mathrm{sgn}\left(x\right)\cdot |x{|}^{\alpha }\cdot \chi_{max},\;\;\;P_{optimized}=P_{0}+\Delta P$$

where α≥1.5 is a sensitivity factor. The introduction of sgn(·) significantly stretches the search space near the initial expert value $P_{0}$, thereby improving the search resolution in that region. As the deviation increases, the mapping step size $\chi_{max}$ grows exponentially, ensuring that the algorithm retains the ability to escape local optima even far from the expert’s experience.

Step 3: Projection and constraint handling.

Candidate solutions are orthogonally projected onto the feasible region to ensure that all parameters satisfy the constraints.

Step 4: Objective function evaluation.

The objective function is defined as

(29) $$\min_{P}T\left(P\right)=\left(1-f_{acc}\left(\alpha_{c},r,w,v\right)\right)+\lambda \sum_{i=1}^{N}{∥\frac{P_{i}-P_{0,i}}{\sigma_{i}}∥}^{2}\;\;\;s.t.\left\{\begin{array}{c} 0\leq r,\\ \alpha_{c}\in \left(l,u\right),\\ 0\leq w\leq 1,\\ 0\leq v\leq 1 \end{array}\right.$$

where $f_{acc}$ is the diagnostic accuracy of the model, serving as the primary driving term. The second term is a knowledge deviation penalty, with λ being a penalty coefficient that prevents the optimized parameters from deviating excessively from the initial values $P_{0}$ provided by experts; $\sigma_{i}$ represents the search weight for the corresponding parameter.

Step 5: Recording the optimal solution.

Candidate solutions are sorted in ascending order of the objective function value, and the optimal solution is recorded.

Step 6: Mean update guided by dynamic knowledge anchoring.

At the level of evolutionary intervention, the parallel search capability of P-CMA-ES is leveraged to guide the evolutionary paths of subpopulations using dynamic knowledge anchoring. In each generation, the update of the distribution mean $m_{g+1}$ no longer depends solely on the weighted average of the current generation’s elite individuals. Instead, a dynamically decaying attraction term $\eta_{g}P_{0}$ is introduced. The evolutionary center $m_{g+1}$ is calculated as

(30) $$m_{g+1}=\left(1-\eta_{g}\right)\sum_{i=1}^{\mu }\theta_{i}\gamma_{i:g+1}+\eta_{g}P_{0}$$

(31) $$\eta_{g}=\eta_{start}{\left(1-\frac{g}{G_{\max}}\right)}^{k}$$

where $\eta_{g}$ is the knowledge anchoring strength, $\theta_{i}$ denotes the recombination weights, $\gamma_{i:g+1}$ represents the elite individual of the *i*th generation, *g* is the current generation number, $G_{max}$ is the maximum number of generations, and *k* is the decay exponent. This strategy forces the algorithm to intensively search within the physically acceptable bounds defined by expert knowledge during the early stages of optimization. With the advancement of iteration, the strength of knowledge anchoring gradually decreases, so that the algorithm obtains stronger data-driven correction ability. At the same time, the parameter deviation penalty term still prevents the optimization parameters from deviating unconstrained from expert knowledge.

Step 7: Updating the evolution paths and covariance matrix.

Based on the mean shift, the step-size evolution path and the covariance evolution path are updated. The covariance matrix and step size are then updated accordingly, and eigendecomposition is performed periodically to maintain numerical stability.

Step 8: Output.

After reaching the maximum number of generations, the optimal solution $P^{*}$ and its corresponding diagnostic accuracy $f_{acc}\left(P^{*}\right)$ are obtained.

**Remark 3.** 
*Dynamic knowledge anchoring is employed to alleviate the issue of parameters deviating from expert knowledge and physical meanings during the data-driven optimization process. Unlike optimization methods that solely pursue diagnostic accuracy, this mechanism introduces expert knowledge constraints into the optimization process, enabling the model to enhance performance while maintaining the interpretability of rule parameters.*

## 5. Case Study

**Figure 3:**
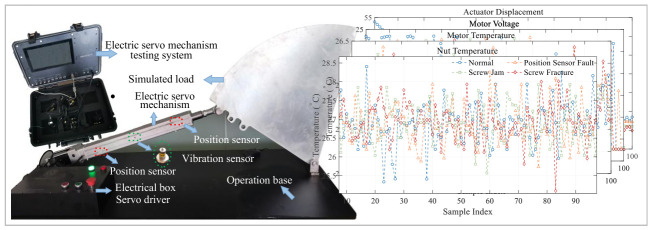
EMA fault experiment platform.

To validate the effectiveness and accuracy of the proposed fault diagnosis method, this section presents data collection and algorithm validation using an electromechanical actuator (EMA) fault experiment platform. As the core execution terminal of modern complex electromechanical equipment, the operational reliability of the EMA directly dictates the safety and stability of the entire system. As shown in Figure 3, the platform consists of a test system, a driver, an EMA, and a simulated load. By deliberately injecting faults into the platform, a raw dataset covering multiple operating conditions is obtained, providing support for model training and validation.

In complex industrial or aeronautical applications, EMAs often operate under harsh conditions such as variable loads and reciprocating motion at high frequency, making mechanical wear and electrical aging inevitable. In this experiment, four typical working states were defined based on common EMA failure mechanisms: normal operation ($F_{1}$), position sensor fault ($F_{2}$), screw jamming ($F_{3}$), and screw fracture ($F_{4}$). A total of 1000 data samples were collected and divided into training and test sets with an 8:2 ratio. A position sensor fault mainly affects the feedback signal of actuator displacement and may lead to abnormal control compensation in motor voltage, while the thermal features are usually affected indirectly. Screw jamming increases mechanical resistance during transmission, which can induce abnormal motor voltage and a temperature rise in the screw–nut pair due to frictional heating. Screw fracture weakens or interrupts the mechanical transmission path, resulting in abnormal displacement response and inconsistent thermal or electrical characteristics. Based on these mechanisms, the actuator displacement ($x_{1}$), motor voltage ($x_{2}$), motor temperature ($x_{3}$), and nut temperature ($x_{4}$) were selected as the core fault feature vector. In constructing the reference grades of fault features, the experiments in this paper adopt a three-point reference value setting method based on sample statistical characteristics. For each fault feature, its value range is counted in the sample space, and the lower bound, mean, and upper bound of the experimental sample space of the feature are extracted, respectively, as the reference values of the three reference grades of low, medium, and high, namely *L*, *M*, and *H*. Among them, *L* represents the low value level of the feature in the sample space, *H* represents the high value level of the feature in the sample space, and *M* represents the medium value level of the feature. Reference levels and values for each feature were defined, as shown in Table 3.

In the traditional BRB modeling process, experts directly provide the initial rule base. In this experimental case, the initial rule base contains 81 rules, requiring the filling of 324 parameters based on the mapping relationship between fault features and diagnostic results. The large number of parameters makes it difficult to assign accurate confidence levels to the rules. This paper establishes an interval grey number table, a form of information transformation that not only preserves the uncertainty of expert knowledge but also significantly reduces the number of parameters and lowers the difficulty of mapping, requiring only 48 interval grey numbers. As shown in Table 4, the interval grey number table provides a priori knowledge foundation for EMA fault diagnosis.

Following the transformation process described in Section 3, the BRB-IG fault diagnosis model is established. In order to further explain how the expert interval grey judgment is converted into the initial belief rule, the first rule is taken as an example. The initial fault feature weights and attribute weights are all set to 1. The antecedent of this rule is $x_{1}=L$, $x_{2}=L$, $x_{3}=L$, and $x_{4}=L$. According to the interval grey number table in Table 4, the expert judgments corresponding to this antecedent combination under four fault modes are extracted. $x_{1}=L$ corresponding to $F_{1}-F_{4}$ are, respectively, [0.4, 0.7], [0, 0.4], [0.1, 0.6], and [0.2, 0.7]. For $x_{2}=L$, the corresponding interval grey numbers are respectively [0.1, 0.3], [0.2, 0.6], [0.4, 0.6], and [0.1, 0.5]. For $x_{3}=L$, the corresponding interval grey numbers are, respectively, [0, 1], [0, 0.3], [0, 0.3], and [0.1, 0.4]. For $x_{4}=L$, the corresponding interval grey numbers are, respectively, [0.5, 0.8], [0.2, 0.4], [0, 0.3], and [0.1, 0.6]. According to Equations (7)–(15), the whitened value and certainty degree of each interval grey number are calculated respectively, and four pieces of evidence extracted are obtained:

(32) $$\begin{array}{cc} \; & E_{1,1}:\mathrm{If}\;x_{1}\;\mathrm{is}\;L,\;\mathrm{then}\;\left\{\left(F_{1},0.3257\right),\left(F_{2},0.2148\right),\left(F_{3},0.2157\right),\left(F_{4},0.2439\right)\right\}\\ \; & \;\;\;\;\;\;\;\;\;\mathrm{with}\;\mathrm{Confidence}\;\{0.9048,0.6742,0.6478,0.6798\}\;\mathrm{and}\;\mathrm{fault}\;\mathrm{feature}\;\mathrm{weight}\;1 \end{array}$$

(33) $$\begin{array}{cc} \; & E_{2,1}:\mathrm{If}\;x_{2}\;\mathrm{is}\;L,\;\mathrm{then}\;\left\{\left(F_{1},0.1714\right),\left(F_{2},0.2576\right),\left(F_{3},0.3665\right),\left(F_{4},0.2045\right)\right\}\\ \; & \;\;\;\;\;\;\;\;\;\mathrm{with}\;\mathrm{Confidence}\;\{0.9453,0.8344,0.8930,0.8711\}\;\mathrm{and}\;\mathrm{fault}\;\mathrm{feature}\;\mathrm{weight}\;1 \end{array}$$

(34) $$\begin{array}{cc} \; & E_{3,1}:\mathrm{If}\;x_{3}\;\mathrm{is}\;L,\;\mathrm{then}\;\left\{\left(F_{1},0.3947\right),\left(F_{2},0.1688\right),\left(F_{3},0.1627\right),\left(F_{4},0.2711\right)\right\}\\ \; & \;\;\;\;\;\;\;\;\;\mathrm{with}\;\mathrm{Confidence}\;\{0.5963,0.8155,0.8047,0.6939\}\;\mathrm{and}\;\mathrm{fault}\;\mathrm{feature}\;\mathrm{weight}\;1 \end{array}$$

(35) $$\begin{array}{cc} \; & E_{4,1}:\mathrm{If}\;x_{4}\;\mathrm{is}\;L,\;\mathrm{then}\;\left\{\left(F_{1},0.3671\right),\left(F_{2},0.2045\right),\left(F_{3},0.0835\right),\left(F_{4},0.3450\right)\right\}\\ \; & \;\;\;\;\;\;\;\;\;\mathrm{with}\;\mathrm{Confidence}\;\{0.5519,0.3074,0.1255,0.5186\}\;\mathrm{and}\;\mathrm{fault}\;\mathrm{feature}\;\mathrm{weight}\;1 \end{array}$$

After that, according to Equations (19)–(21), the first rule is obtained:

(36) $$\begin{array}{cc} \; & R_{1}:\mathrm{If}\;x_{1}\;\mathrm{is}\;L\wedge x_{2}\;\mathrm{is}\;L\wedge x_{3}\;\mathrm{is}\;L\wedge x_{4}\;\mathrm{is}\;L,\\ \; & \mathrm{then}\;\left\{\left(F_{1},0.2719\right),\left(F_{2},0.2213\right),\left(F_{3},0.1211\right),\left(F_{4},0.3857\right)\right\}\\ \; & \mathrm{with}\;\mathrm{rule}\;\mathrm{weight}\;\omega_{1}\;\mathrm{and}\;\mathrm{fault}\;\mathrm{feature}\;\mathrm{weight}\;\varepsilon_{1},\varepsilon_{2},\varepsilon_{3},\varepsilon_{4} \end{array}$$

Partial initial rules are presented in Table 5.

**Figure 4:**
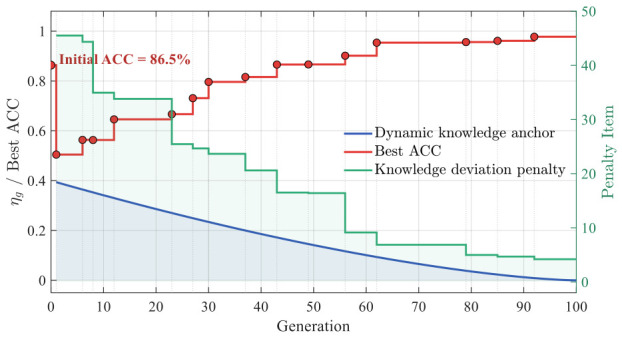
The process of the optimization method based on dynamic knowledge anchoring.

To further improve the diagnostic accuracy, the dynamic knowledge anchoring optimization method is applied to adjust the model parameters. The optimization iteratively refines the interval grey numbers, rule weights, and fault feature weights to reduce the gap between subjective experience and objective reality. Figure 4 illustrates the co-evolution process of the dynamic knowledge anchoring strength $\eta_{g}$, the parameter distance penalty P, and the best diagnostic accuracy across iterations. Prior to the optimization, the initial model accuracy, which relies entirely on expert experience, reaches 86.5%. This indicates that the interval grey numbers and initial rules provided by experts lay a solid baseline for the model. During the first optimization iteration, as the algorithm attempts to deviate from the initial expert rules to explore a new parameter space, the ACC of the model experiences a temporary decline. As the iterations progress, the dynamic knowledge anchoring strength $\eta_{g}$ exhibits a smooth and continuous exponential decay. This decay mechanism gradually relaxes the rigid constraints of expert knowledge on the model. Accompanied by the decline in $\eta_{g}$, the model discovers superior feature mapping relationships under the guidance of data, and the parameter distance penalty decreases significantly. After approximately 90 iterations, the accuracy converges to 97.75%.

In order to elucidate the relationship between the preservation of interpretability and the enhancement of optimization performance, Table 6 summarizes the key indicators at representative iteration steps during the dynamic knowledge anchoring optimization process, including the anchoring strength, parameter distance penalty, diagnostic accuracy, and expert knowledge consistency. Among them, the expert knowledge consistency is measured by the ratio of the intersection of the interval grey numbers before and after optimization to the optimized measure, thereby quantifying the extent to which the optimized parameters fall within the scope of expert knowledge, and is defined as

(37) $$C_{expert}=\frac{1}{N\times M\times J_{M}}\sum_{n=1}^{N}\sum_{m=1}^{M}\sum_{i=1}^{J_{M}}\frac{O_{length}\left(⊗g_{opt,m,i}^{n},⊗g_{0,m,i}^{n}\right)}{w_{opt,m,i}^{n}}$$

The range of this indicator is [0,1]. $⊗g_{0,m,i}^{n}$ denotes the expert’s initial interval grey number, and $⊗g_{opt,m,i}^{n}$ denotes the optimized interval grey number. When $C_{expert}=1$, it indicates that the optimized interval is entirely situated within the range of the expert’s initial interval. When $0<C_{expert}<1$, it implies that the optimization results exceed the boundaries of the expert’s initial knowledge.

Table 6 indicates that during the early stage of optimization, since the model is still in the search and exploration phase, the diagnostic accuracy exhibits certain fluctuations; the diagnostic accuracy of the 20th generation is lower than that of the initial model. This phenomenon demonstrates that the optimization algorithm needs to explore a larger parameter space in the early stage, and it does not imply a degradation in the performance of the final model. As the iteration process progresses, the dynamic knowledge anchoring intensity gradually decays from 0.4000 to 0, allowing the model to progressively gain stronger data-driven adjustment capabilities, and the diagnostic accuracy increases from the initial 0.8650 to the final 0.9775. Concurrently, the parameter distance penalty term decreases overall, and the expert knowledge consistency gradually improves from 0.5164 to 0.9455, indicating that the optimized interval grey numbers are progressively maintained within the reasonable range defined by expert knowledge. Therefore, the proposed dynamic knowledge anchoring strategy does not solely pursue the enhancement of diagnostic accuracy; rather, it achieves a good balance between diagnostic performance enhancement and interpretability preservation.

On this basis, three evaluation indexes including average uncertainty, information compression rate and expert knowledge consistency are introduced to quantitatively analyze the optimization results. The average uncertainty degree is calculated based on the comprehensive uncertainty measure defined in Section 3.2 and is used to measure the comprehensive uncertainty of the interval grey numbers. The average measure is utilized to reflect the uncertainty range of the interval grey numbers, while the information compression rate characterizes the compression degree of the interval width before and after optimization.

The average measure is defined as

(38) $$\bar{W}=\frac{1}{N\times M\times J_{M}}\sum_{n=1}^{N}\sum_{m=1}^{M}\sum_{i=1}^{J_{M}}\omega_{m,i}^{n}$$

The information compression rate is defined as

(39) $$ICR=\frac{{\bar{W}}_{initial}-{\bar{W}}_{optimized}}{{\bar{W}}_{initial}}\times 100\%$$

where ${\bar{W}}_{initial}$ represents the average measure of the initial interval grey numbers provided by experts before optimization, and ${\bar{W}}_{optimized}$ represents the average measure of the optimized interval grey numbers. A larger ICR indicates a more significant compression of the uncertainty range of the optimized interval grey numbers.

**Figure 5:**
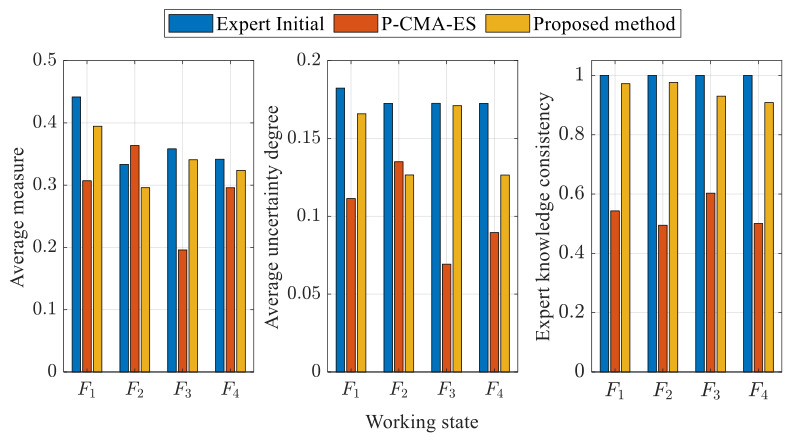
Quantitative comparison of interval grey number optimization results under different fault types.

Table 7 presents the overall quantitative comparison results before and after the optimization of interval grey numbers. Figure 5 further illustrates the grouped comparison results of the average interval width, average uncertainty degree, and expert knowledge consistency according to different fault types.

The results indicate that although the P-CMA-ES heuristic search method can achieve an optimized average uncertainty of 0.1021 and an information compression rate of 21.18%, its expert knowledge consistency is only 0.53544. This implies that after optimization, many intervals still exceed or deviate from the expert’s initial knowledge boundaries, presenting a certain risk of knowledge distortion. In contrast, the optimization method proposed in this paper reduces the average uncertainty to 0.1371 and the average measure to 0.3312, with an information compression rate of 10.17%, effectively reducing uncertainty and achieving moderate compression. More importantly, the expert knowledge consistency reaches 0.9455, which is significantly higher than that of P-CMA-ES, indicating that the proposed method essentially preserves the expert knowledge boundaries during the optimization process. Analyzed by fault type, the expert knowledge consistency of the proposed method is maintained at a high level across all four operating states, while the average measures and uncertainties for the four types of faults have also decreased. This demonstrates that the proposed dynamic knowledge anchoring strategy does not merely pursue compression but rather achieves a better balance among reducing uncertainty, compressing the interval range, and preserving expert knowledge consistency.

**Figure 6:**
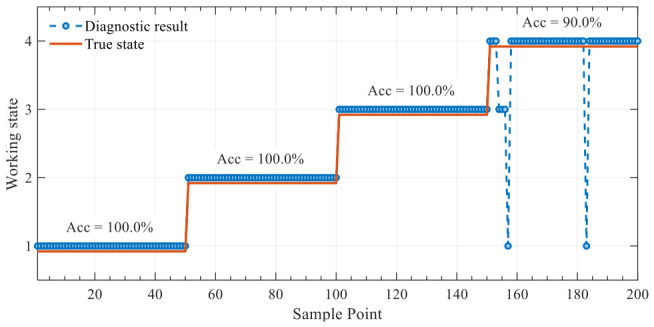
Diagnostic results of the model on the test set.

With the above optimization, the established EMA fault diagnosis model achieves strong performance on the test set. As shown in Figure 6, the recognition accuracy reaches 100% for $F_{1}$, $F_{2}$, and $F_{3}$, demonstrating precise diagnostic capability. For $F_{4}$, the accuracy is 90%, with only a few false negatives. The overall diagnostic accuracy is 97.5%.

In order to further verify the necessity of interval grey number representation, certainty-weighted fusion, and dynamic knowledge anchoring mechanism in the proposed framework, this paper designs progressive ablation experiments, and the results are shown in Table 8. Specifically, five sets of comparison models are constructed:

(1) M1: This model is without interval grey numbers, without certainty degree, and without optimization. This model replaces the interval judgments given by experts with interval midpoint values and no longer retains the upper and lower bound information in expert judgments, which is used to test the role of interval grey number representation relative to point-valued expert knowledge.

(2) M2: This model introduces interval grey number representation but does not introduce certainty degree and optimization. By comparing M1 and M2, the contribution of interval grey number representation in retaining the boundary information of linguistic fuzzy knowledge and improving diagnostic performance can be analyzed.

(3) M3: This model introduces interval grey number representation and certainty-weighted evidence fusion but does not perform parameter optimization. It is used to verify whether certainty-weighted evidence fusion can reduce the impact of unreliable evidence on diagnostic results.

(4) M4: This model employs P-CMA-ES optimization on the basis of M3. This model is used to analyze the impact of pure data-driven optimization on diagnostic performance improvement and parameter deviation from expert knowledge.

(5) M5: This model employs the dynamic knowledge anchoring optimization method proposed in this paper on the basis of M3. This model is the complete model, used to verify whether the dynamic knowledge anchoring mechanism can reduce the deviation of parameters from expert knowledge and physical meanings while improving the diagnostic performance, thereby maintaining model interpretability.

As can be seen from Table 8, with the step-by-step introduction of interval grey number representation, certainty-weighted fusion, and the dynamic knowledge anchoring mechanism, the overall performance of the model has been continuously improved. Compared with M1, the diagnostic accuracy of M2 increased from 79.0% to 81.5%, indicating that the interval grey number representation can retain the upper and lower bound information in expert judgments and has a stronger capability of uncertain knowledge expression compared with direct point-value processing using interval midpoints. Further, after M3 introduces certainty-weighted fusion on the basis of M2, the accuracy increases to 86.0%, and the macro-average precision reaches 0.8636, indicating that the certainty mechanism can improve the diagnostic performance to a certain extent. M4 achieves an accuracy of 98.0% after using P-CMA-ES optimization, but its expert knowledge consistency is only 0.5354. In contrast, the proposed model M5 in this paper increases the expert knowledge consistency to 0.9455 while maintaining a high accuracy of 97.5%, indicating that the dynamic knowledge anchoring mechanism can effectively constrain the parameter optimization direction and maintain model interpretability under the condition of maintaining better diagnostic performance. In summary, the ablation experiment results verify the necessity of interval grey number representation, certainty-weighted fusion, and dynamic knowledge anchoring mechanism from both aspects of diagnostic performance and expert knowledge consistency.

To verify the stability and generalization ability of the proposed method, comparative experiments are carried out under different parameter configurations. Taking the original interval grey numbers given by experts as the baseline, minor proportional scaling is performed on the interval radius to test the model’s stability against fluctuations in expert prior knowledge. To clearly analyze the independent influence of each key parameter on model performance, a single-factor sensitivity analysis method is adopted based on the original model parameter configuration as the baseline, setting different interval grey numbers, dynamic knowledge anchoring intensities, and penalty coefficients. Each group of experiments is repeated 10 times, and the average values are taken. The experimental results are shown in Table 9.

As can be seen from Table 9, within the set range of parameter perturbations, the BRB-IG model maintains a high diagnostic performance overall. Compared with the baseline, the accuracy, precision, recall, and F1-score of each experimental group exhibit only minor fluctuations without any significant decrease, indicating that the model possesses good stability against changes in key parameters. When the radius of the interval grey numbers given by experts is narrowed to 0.9 times or expanded to 1.1 times, the model performance changes minimally, demonstrating that the proposed method is insensitive to uncertainty perturbations of the expert prior intervals. When the initial anchoring intensity and penalty coefficient vary within a reasonable range, all metrics of the model remain stable as well. Among them, the model achieves the optimal performance under the baseline parameter configuration, indicating that appropriate knowledge anchoring and penalty constraints contribute to enhancing the model’s diagnostic effect and stability. The experiments indicate that the BRB-IG model does not depend on a specific fixed parameter combination, and it can still maintain a stable diagnostic performance when minor changes occur in the grey interval radius, initial anchoring intensity, and penalty coefficient, further verifying the stability and engineering applicability of the proposed method.

**Figure 7:**
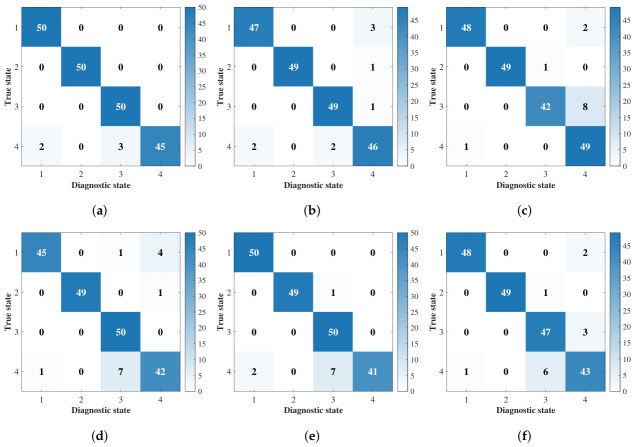
Results of comparative experiments on the test set. (**a**) The result of BRB-IG. (**b**) The result of SVM. (**c**) The result of Adaboost. (**d**) The result of KNN. (**e**) The result of K-means. (**f**) The result of CART.

To further verify the performance of the proposed model, multiple comparative experiments were designed. Support Vector Machine (SVM), Adaptive Boosting (AdaBoost), K-means clustering, K-Nearest Neighbors (KNN), and Classification and Regression Tree (CART) were selected as baseline comparative algorithms. All algorithms were independently executed 10 times on the identical test set, and the optimal diagnostic results were recorded for comparative analysis. Furthermore, the confusion matrices in Figure 7 intuitively illustrate the classification performance of each model across different fault modes, while Table 10 compares the fault diagnosis performance of the algorithms across four evaluation metrics: overall accuracy, precision, recall, and F1-score.

Compared with the classical machine learning models, the BRB-IG model achieves the best performance across multiple evaluation metrics. Moreover, its confusion matrix shows a more concentrated diagonal distribution and fewer misclassified samples. This indicates that BRB-IG has better discriminative ability and classification balance among fault categories. The underlying reason is that the proposed method effectively integrates expert knowledge with data-driven information, demonstrating strong robustness and adaptability when dealing with uncertainty, nonlinear characteristics, and similarities among fault classes in the fault samples.

## 6. Conclusions

This paper focuses on the effective utilization of expert knowledge in fault diagnosis and proposes an interpretable belief rule base method that integrates interval grey number representation, evidence fusion based on certainty degree, and dynamic knowledge anchoring optimization. The use of interval grey numbers preserves the uncertainty inherent in expert knowledge, providing a mathematical foundation for constructing complete rules. The introduction of certainty degree overcomes the variability in cognition across different faults and mitigates the influence of low confidence evidence during fusion. Additionally, a dynamic knowledge anchoring optimization method is proposed. By imposing interpretability constraints during the optimization process, the model is ensured to align with engineering common sense and physical meaning. In summary, this method provides an interpretable solution for fault diagnosis in complex electromechanical equipment that not only makes full use of expert experience but also leverages historical data to achieve reliable and interpretable optimization.

In future research, this method will be extended to fault diagnosis with multi-modal data fusion, integrating heterogeneous information from multiple sources to improve diagnostic accuracy. Meanwhile, to address the potential occurrence of unknown faults in practical engineering, attempts will be made to equip the model with the ability to recognize novel fault types by utilizing residual belief degrees in the belief rule base or designing specific uncertainty measures. This would avoid the forced classification of unseen faults and further enhance the reliability of the system under extremely complex operating conditions.

## Figures and Tables

**Table 1:** Comparison between existing rule-based reasoning frameworks and the proposed method.

Aspect	Fuzzy Rule-Based Reasoning Methods	Conventional BRB	Proposed Method
Knowledge input format	Typically employs fuzzy membership degrees to express expert knowledge.	Adopts IF-THEN rules with belief distributions, representing rule consequents as multiple assessment grades and their corresponding belief degrees.	Expresses linguistic fuzzy judgments given by experts as interval grey numbers, used to characterize the uncertainty of expert knowledge.
Initial rule construction approach	Mainly relies on experts to set the rules.	Experts preset the antecedent reference levels, rule combinational structures, and initial belief degrees of consequents.	Performs evidence extraction and fusion based on interval grey numbers and a certainty degree mechanism to generate initial belief rules.
Knowledge uncertainty handling	Mainly manifested as fuzzified inputs.	Mainly describes the uncertainty of rule conclusions through the consequent belief degree distribution.	Utilizes interval grey numbers to reflect the comprehensive uncertainty of knowledge and introduces certainty degree into the evidence fusion process to characterize the differences in the reliability of expert knowledge under different fault modes.
Parameter optimization scheme	Does not involve parameter optimization.	Usually takes improving diagnostic accuracy as the primary objective, optimizing rule weights, fault feature weights, and belief degrees.	Optimizes interval grey numbers, rule weights, and fault feature weights and introduces a dynamic knowledge anchoring mechanism into the parameter optimization process to constrain the deviation of optimized parameters from expert knowledge.

**Table 2:** Interval grey number table.

Fault Feature	$x_{1}$	$x_{2}$	⋯	$x_{M}$
Reference Value	$\left(A_{1}^{1},A_{1}^{2},\ldots ,A_{1}^{J_{1}}\right)$	$\left(A_{2}^{1},A_{2}^{2},\ldots ,A_{2}^{J_{2}}\right)$	⋯	$\left(A_{M}^{1},A_{M}^{2},\ldots ,A_{M}^{J_{M}}\right)$
$F_{1}$	$\left(⊗g_{1,1}^{1},\cdots ,⊗g_{1,J_{1}}^{1}\right)$	$\left(⊗g_{2,1}^{1},\ldots ,⊗g_{2,J_{2}}^{1}\right)$	⋯	$\left(⊗g_{M,1}^{1},\ldots ,⊗g_{M,J_{M}}^{1}\right)$
$F_{2}$	$\left(⊗g_{1,1}^{2},\ldots ,⊗g_{1,J_{1}}^{2}\right)$	$\left(⊗g_{2,1}^{2},\ldots ,⊗g_{2,J_{2}}^{2}\right)$	⋯	$\left(⊗g_{M,1}^{2},\ldots ,⊗g_{M,J_{M}}^{2}\right)$
⋯	⋯	⋯	⋯	⋯
$F_{N}$	$\left(⊗g_{1,1}^{N},\ldots ,⊗g_{1,J_{1}}^{N}\right)$	$\left(⊗g_{2,1}^{N},\ldots ,⊗g_{2,J_{2}}^{N}\right)$	⋯	$\left(⊗g_{M,1}^{N},\ldots ,⊗g_{M,J_{M}}^{N}\right)$

**Table 3:** Fault feature reference level and its reference value.

Fault Feature	Reference Value		
	L	M	H
$x_{1}$	23.666583	31.19726592	78.031353
$x_{2}$	−25.117308	−4.135547491	23.48679
$x_{3}$	18.157165	32.4322707	46.368845
$x_{4}$	23.274949	30.51674331	44.016766

**Table 4:** Table of interval grey numbers for EMA.

Fault Feature	$x_{1}$			$x_{2}$		
Reference Level	L	M	H	L	M	H
$F_{1}$	[0.4, 0.7]	[0.1, 0.6]	[0.2, 0.5]	[0.1, 0.3]	[0.1, 0.7]	[0.2, 0.9]
$F_{2}$	[0, 0.4]	[0, 0.5]	[0, 0.5]	[0.2, 0.6]	[0.6, 0.9]	[0.1, 0.3]
$F_{3}$	[0.1, 0.6]	[0.1, 0.8]	[0.3, 0.7]	[0.4, 0.6]	[0.2, 0.7]	[0, 0.4]
$F_{4}$	[0.2, 0.7]	[0.2, 0.5]	[0.4, 0.8]	[0.1, 0.5]	[0.4, 0.8]	[0.3, 0.6]
**Fault Feature**	$x_{3}$			$x_{4}$		
**Reference Level**	**L**	**M**	**H**	**L**	**M**	**H**
$F_{1}$	[0, 1]	[0, 0.3]	[0.1, 0.5]	[0.5, 0.8]	[0, 0.4]	[0.1, 0.4]
$F_{2}$	[0, 0.3]	[0, 0.2]	[0.5, 0.8]	[0.2, 0.4]	[0.2, 0.7]	[0.1, 0.4]
$F_{3}$	[0, 0.3]	[0.4, 0.8]	[0.1, 0.3]	[0, 0.3]	[0.5, 0.8]	[0.6, 0.7]
$F_{4}$	[0.1, 0.4]	[0.1, 0.5]	[0.1, 0.4]	[0.5, 0.8]	[0.1, 0.6]	[0.1, 0.3]

**Table 5:** Partial initial rules.

*k*	$x_{1}\wedge x_{2}\wedge x_{3}\wedge x_{4}$	$\left\{\xi_{1,k},\xi_{2,k},\xi_{3,k},\xi_{4,k}\right\}$
3	L∧L∧L∧H	{0.0872,0.1095,0.2726,0.5307}
13	L∧M∧M∧L	{0.1754,0.0901,0.2339,0.5005}
23	L∧H∧M∧M	{0.1615,0.1642,0.3075,0.3667}
33	M∧L∧M∧H	{0.0664,0.2901,0.4543,0.1892}
43	M∧M∧H∧L	{0.2729,0.2451,0.1448,0.3372}
53	M∧H∧H∧M	{0.2215,0.3931,0.1673,0.2181}
63	H∧L∧H∧H	{0.0706,0.4647,0.2525,0.2122}
73	H∧H∧L∧L	{0.4194,0.2066,0.1050,0.2689}
81	H∧H∧H∧H	{0.1792,0.4642,0.1780,0.1786}

**Table 6:** Evolution of key indicators during the optimization process.

Iteration	Anchoring Strength	Parameter Distance Penalty	Diagnostic Accuracy	Expert Knowledge Consistency
0	0.4	—	0.8650	1
20	0.2862	45.5089	0.6261	0.5164
40	0.1859	33.8114	0.8660	0.7502
60	0.1012	16.5109	0.9015	0.8475
80	0.0355	9.4360	0.9538	0.9123
100	0	5.1852	0.9775	0.9455

**Table 7:** Quantitative comparison before and after the optimization of interval grey numbers.

Scheme	Average Uncertainty Degree	Average Measure	Information Compression Rate	Expert Knowledge Consistency
BRB-IG	0.1750	0.3687	—	1.000
P-CMA-ES optimization method	0.1021	0.2906	21.18%	0.5354
The proposed optimization method	0.1371	0.3312	10.17%	0.9455

**Table 8:** Ablation study of the proposed components.

Model	ACC	Precision	Recall	F1	Expert Knowledge Consistency
M1	79.0%	0.7910	0.7900	0.7900	—
M2	81.5%	0.8156	0.8150	0.8151	—
M3	86.0%	0.8636	0.8600	0.8603	—
M4	98.0%	0.9803	0.9800	0.9800	0.5354
M5	97.5%	0.9762	0.9750	0.9747	0.9455

**Table 9:** Sensitivity analysis under different parameter configurations.

Experimental Group	Gray Interval Radius Ratio	Initial Anchoring Strength	Coefficient of Penalty	ACC	Precision	Recall	F1
Baseline	1.0	0.4	0.25	97.50%	0.9762	0.9750	0.9747
Gray interval A	0.9	0.4	0.25	96.00%	0.9606	0.9600	0.9598
Gray interval B	1.1	0.4	0.05	96.15%	0.9623	0.9615	0.9617
Anchoring strength A	1.0	0.3	0.25	96.45%	0.9646	0.9645	0.9645
Anchoring strength B	1.0	0.5	0.25	96.00%	0.9604	0.9600	0.9600
Coefficient of penalty A	1.0	0.4	0.2	95.50%	0.9551	0.9550	0.9550
Coefficient of penalty B	1.0	0.4	0.3	95.75%	0.9576	0.9575	0.9575

**Table 10:** Comparison of model evaluation metrics.

Model	Accuracy	Precision	Recall Rate	F1 Score
BRB-IG	97.50%	0.9762	0.9750	0.9747
SVM	95.50%	0.9555	0.9550	0.9552
Adaboost	94.00%	0.9467	0.9400	0.9433
KNN	93.00%	0.9335	0.9300	0.9317
K-means	95.00%	0.9559	0.9500	0.9529
CART	93.50%	0.9364	0.9350	0.9357

## Data Availability

The data presented in this study are available on request from the corresponding author. The data are not publicly available due to project-related restrictions.
